# NMR resonance assignments of a hypoallergenic isoform of the major birch pollen allergen Bet v 1

**DOI:** 10.1007/s12104-017-9754-7

**Published:** 2017-08-14

**Authors:** Linda Ahammer, Sarina Grutsch, Michael Wallner, Fatima Ferreira, Martin Tollinger

**Affiliations:** 1 Institute of Organic Chemistry, Center for Molecular Biosciences Innsbruck (CMBI), University of Innsbruck, Innrain 80/82, 6020 Innsbruck, Austria; 2 Department of Molecular Biology, University of Salzburg, Hellbrunnerstraße 34, 5020 Salzburg, Austria

**Keywords:** NMR resonance assignment, Bet v 1, Birch, Allergen

## Abstract

In Northern America and Europe a great number of people are suffering from birch pollen allergy and pollen related food allergies. The trigger for these immunological reactions is the 17.5 kDa major birch pollen allergen Bet v 1, which belongs to the family of PR-10 (pathogenesis-related) proteins. In nature, Bet v 1 occurs as a mixture of various isoforms that possess different immunological properties despite their high sequence identities. Bet v 1.0102 (Bet v 1d), which is investigated here, is a hypoallergenic isoform of Bet v 1 and a potential candidate for allergen-specific immunotherapy. We assigned the backbone and side chain ^1^H, ^13^C and ^15^N resonances of this protein and predicted its secondary structure. The NMR-chemical shift data indicate that Bet v 1.0102 is composed of three α-helices and a seven stranded β-sheet, in agreement with the known structure of the hyperallergenic isoform Bet v 1.0101 (Bet v 1a). Our resonance assignments create the foundation for detailed characterization of the dynamic properties of Bet v 1 isoforms by NMR relaxation measurements.

## Biological context

Bet v 1 is a highly immunogenic protein that represents the main cause for allergic sensitization against birch pollen (Ipsen and Lowenstein 1983). In the temperate climate zone of the northern hemisphere, an estimated 100 million people are allergic to birch pollen, up to 90% of individuals that are sensitized to birch pollen exhibit serum IgE reactivity towards Bet v 1 (Moverare et al. 2002). This allergen belongs to the family of pathogenesis-related proteins of class 10 (PR-10), which are found in a multitude of different plants (Fernandes et al. 2013). The exact function of these proteins is still unknown, but a key role in defence of plants is assumed, as they are encoded by genes that are activated in response to different kinds of abiotic and biotic stress.

Naturally occurring Bet v 1 is a composition of different isoforms with a molecular mass around 17.5 kDa, which share high sequential identities but can have a drastically different allergenic potential (Ferreira et al. 1996). To date more than twenty isoforms are listed by the IUIS Allergen Nomenclature Sub-Committee (http://www.allergen.org). With 35% of total Bet v 1 in grained pollen, the hyperallergenic isoform Bet v 1.0101 (Bet v 1a) is the most abundant one (Ferreira et al. 1996). Experiments with sera of birch pollen allergic individuals showed that Bet v 1.0101 has the ability to induce a high level of IgE antibody production, which results in type I allergic reactions (Wagner et al. 2008). This is contrasted by Bet v 1.0102 (Bet v 1d), which possesses a sequence identity of 95.6% to Bet v 1.0101 but activates IgG4 expression (Wagner et al. 2008). The hyperallergenic isoform Bet v 1.0101 thus plays an important role for allergic sensitization, while the hypoallergenic Bet v 1.0102 initiates the defending immune response. Furthermore, hypoallergenic isoforms are potential candidates for allergen-specific immunotherapy (Valenta et al. 2016). Very recent studies hypothecate that these different immunological properties may result from a varying dynamical behavior and fold stabilities (Freier et al. 2015; Machado et al. 2016).

The three-dimensional structures of numerous Bet v 1 isoforms and mutants are available in the literature (Fernandes et al. 2013). Bet v onefolds into a curved seven-stranded antiparallel β-sheet, along with two short α-helices and an extended C-terminal helix, which create an expanded internal cavity, as it is typical for the PR-10 protein family. Of note, Bet v 1 structures that are available in the protein data base (PDB) exhibit only small differences, with backbone pair-wise rmsd values between 1.5 and 2 Å. For Bet v 1.0102 a three-dimensional structure has not been reported so far. Homologous PR-10 proteins found in fruits and vegetables with similar three-dimensional structures can elicit allergic cross-reactions with Bet v 1-specific IgE antibodies, including apples, cherries, peaches and others (Mills and Shewry 2004).

Here we present the solution NMR backbone and side chain assignment of hypoallergenic Bet v 1.0102 and a chemical shift-derived three-dimensional structure model of this protein created by CS-Rosetta. Chemical shift assignments of Bet v 1 are the prerequisite for future NMR dynamics studies of this protein.

## Methods and experiments

### Protein expression and purification

For recombinant protein production the vector pET28b containing codon-optimized DNA of Bet v 1.0102 was used. The expression and purification protocol was adapted from Wallner et al. (2009). Protein expression in *E. coli* strain BL21 (DE3) Star was carried out in 1 l M9 minimal medium including 25 µg/ml kanamycin enriched with ^15^NH_4_Cl (Cambridge Isotope Laboratories) and ^13^C_6_-d-glucose (Sigma-Aldrich) for isotope labeling at 37 °C. After induction with 0.5 mM IPTG at OD_600_ = 0.6–0.8, the culture was grown overnight at 16 °C and harvested by centrifugation at 2050×*g*. For cell resuspension a buffer containing 25 mM imidazole, 0.1% (v/v) Triton ×100 and 0.5 M urea was used. The cells were lysed by three cycles of freezing (liquid nitrogen) and thawing (water bath at 37 °C). First, DNAse I (1 µg/ml) and after 20 min of shaking, solid NaCl was added to a final concentration of 200 mM. The mixture was centrifuged at 9500×*g* for 30 min and the supernatant was collected. Under constant stirring on ice solid NaCl and NaH_2_PO_4_ were slowly added to a concentration of 1 and 0.5 M, respectively. After 2 h of stirring the precipitated proteins were collected at 9500×*g* for 30 min and the supernatant containing Bet v 1.0102 was filtered through a 45 µm filter. Bet v 1.0102 was purified by hydrophobic interaction chromatography using 3 × 5 ml phenylsepharose columns (HiTrap™ Phenyl FF, GE Healthcare) at a flow rate of 2 ml/min. After the protein solution was loaded, a linear gradient of 30 ml with 0.2 M NaH_2_PO_4_ and 1 M NaCl, pH 4.2 was performed to remove urea. For protein elution, a linear gradient of 180 ml using 25 mM TrisHCl pH 9.3, 8% (v/v) 2-propanol was applied. Bet v 1.0102 containing fractions were collected and concentrated by Amicon Ultra Centrifugal Filters with a cut-off of 3000 Da (Millipore). As final purification step size exclusion chromatography was performed using a 120 ml gel filtration column (HiLoad 16/600 Superdex 75 prep grade, GE Healthcare) with 5 mM sodium phosphate buffer, pH 8.0 at a flow rate of 1 ml/min. An ÄKTAprime™ plus chromatography system (GE Healthcare) was used for all chromatographic steps with an integrated UV-detector for protein detection at 280 nm. The protein concentration was determined using a NanoPhotometer^®^ (Implen). Expression and purification of Bet v 1.0102 were monitored by sodium dodecyl sulfate polyacrylamide gel electrophoresis (SDS–PAGE) using 15% gels. Purified Bet v 1.0102 was dialyzed against 50 mM NaCl, 10 mM sodium phosphate buffer, pH 7.0 for NMR resonance assignment experiments.

### NMR spectroscopy

The following spectra were recorded for NMR resonance assignment on a 500 MHz Agilent DirectDrive spectrometer at 298 K: 2D ^1^H-^15^N-HSQC, ^1^H-^13^C-HSQC, and 3D HNCO, HNCACB, CBCA(CO)NH, (H)CC(CO)NH-TOCSY, H(CC)(CO)NH-TOCSY, and ^1^H^15^N-HSQC-TOCSY. The protein concentration for these NMR measurements was 0.8 mM of ^15^N/^13^C labeled Bet v 1.0102 in 10 mM sodium phosphate, 50 mM NaCl, 90% H_2_O/10% D_2_O (v/v) at pH 7.0. Data processing was done using NMRPipe (Delaglio et al. 1995) and for resonance assignment the program CcpNmr Analysis (Vranken et al. 2005) was used.

### Assignment and secondary structure

**Fig. 1:**
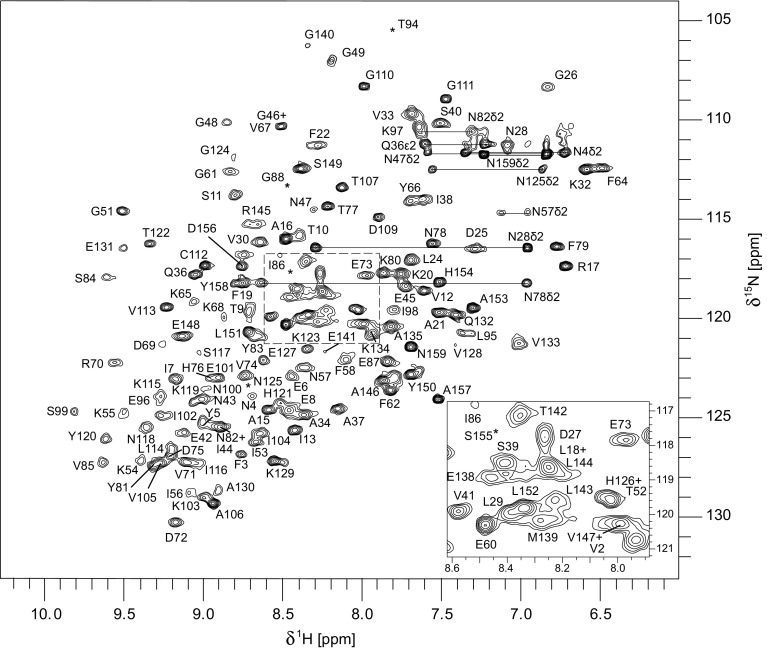
Assigned ^1^H-^15^N-HSQC spectrum of Bet v 1.0102 in 10 mM sodium phosphate, 50 mM NaCl, 10% D_2_O at pH 7.0, 298 K, 500 MHz. NH_2_ side chain resonances are connected by *horizontal lines. Asterisks* indicate the positions of residues below the intensity cut-off

The ^1^H-^15^N-HSQC spectrum of Bet v 1.0102 is shown in Fig. 1. Backbone amide ^1^H-^15^N resonance assignment was obtained for 143 out of 151 non-proline residues, which corresponds to 94.7%. In addition, 96.9% of Cα, 96.9% of Cβ and 94.0% of CO resonances were assigned, and assignment of side chain protons is 87.4% complete. Furthermore, side-chain ^13^C resonances beyond β-positions and side-chain amides in Asn and Glu (^15^N and ^1^H) were assigned partially. The chemical shift data of Bet v 1.0102 have been deposited at the Biological Magnetic Resonance Data Bank (BMRB) with the accession number 27040.

**Fig. 2:**
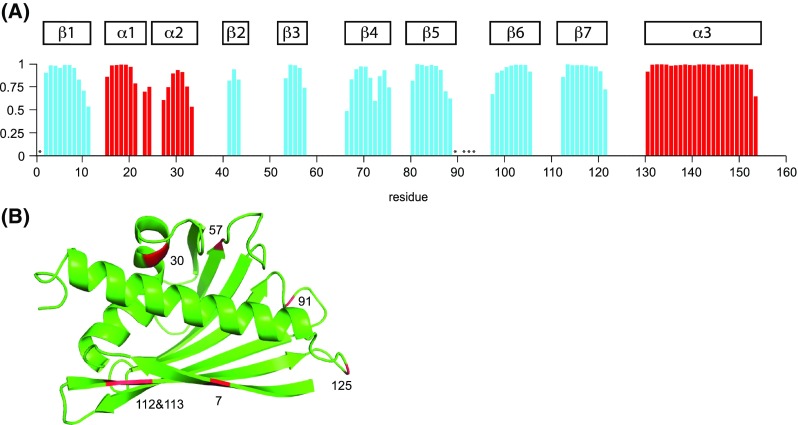
**A** TALOS+ prediction of secondary structure elements of Bet v 1.0102 derived from HN, N, Cα, Cβ and CO chemical shifts. The secondary structure probability is reflected by the *height of the bars* (*blue* β-strands, *red* α-helices). Unassigned backbone amide NH resonance are indicated by *asterisks*. For comparison, the secondary structure elements of Bet v 1.0101 (*α1*–*α3* and *β1*–*β7*) are *shown above*. **B** Lowest energy CS-ROSETTA structure model of Bet v 1.0102. Amino acids that are different in Bet v 1.0102 compared to Bet v 1.0101 are *highlighted in red* (T7I, F30V, S57N, I91V, S112C, I113V, D125N)

Using the Bet v 1.0102 chemical shifts (HN, N, Cα, Cβ and CO), the secondary structure elements of the protein were predicted by TALOS+ (Shen et al. 2009) (Fig. 2a). Our data show that this protein contains seven β-strands (β1−β7) and two short helices (α1−α2), as well as a long C-terminal helix (α3). In addition, a three-dimensional model of the protein was generated using CS-ROSETTA (Lange et al. 2012) (Fig. 2b). In this structure, Bet v 1.0102 consists of a long C-terminal helix that is embraced by a curved, seven-stranded antiparallel β-sheet. Helices α1 and α2 form a V-shaped support for helix α3, creating a large internal cavity, consistent with the canonical PR-10 fold (Fernandes et al. 2013).

Taken together, the Bet v 1.0102 chemical shift data indicate no significant structural difference between the hypoallergenic isoform Bet v 1.0102 and the hyperallergenic isoform Bet v 1.0101 (Gajhede et al. 1996). However, the observed variability in resonance intensities is an indication for the dynamic nature of this protein. A detailed NMR spectroscopic investigation of the dynamic properties of Bet v 1 proteins in the context of their observed immunologic features is currently being performed at our laboratory. In addition, the NMR chemical shift assignments of Bet v 1.0102 build the basis for epitope mapping and ligand binding studies.
